# Comparison of commercial DNA preparation kits for the detection of Brucellae in tissue using quantitative real-time PCR

**DOI:** 10.1186/1471-2334-10-100

**Published:** 2010-04-20

**Authors:** Herbert Tomaso, Mireille Kattar, Meike Eickhoff, Ulrich Wernery, Sascha Al Dahouk, Eberhard Straube, Heinrich Neubauer, Holger C Scholz

**Affiliations:** 1 Friedrich-Loeffler-Institut, Institute of Bacterial Infections and Zoonoses, Naumburgerstrasse 96a, 07743 Jena, Germany; 2 Department of Bacteriology, Bundeswehr Institute of Microbiology, Munich, Germany; 3 Department of Pathology and Laboratory Medicine, American University of Beirut, Beirut, Lebanon; 4 Roche Molecular Diagnostics, Rotkreuz, Switzerland; 5 Central Veterinary Research Laboratory, Dubai, United Arab Emirates; 6 RWTH Aachen University, Department of Internal Medicine III, Aachen, Germany; 7 Institute of Medical Microbiology, Friedrich Schiller University of Jena, Jena, Germany

## Abstract

**Background:**

The detection of Brucellae in tissue specimens using PCR assays is difficult because the amount of bacteria is usually low. Therefore, optimised DNA extraction methods are critical. The aim of this study was to assess the performance of commercial kits for the extraction of *Brucella *DNA.

**Methods:**

Five kits were evaluated using clinical specimens: QIAamp™ DNA Mini Kit (QIAGEN), peqGold™ Tissue DNA Mini Kit (PeqLab), UltraClean™ Tissue and Cells DNA Isolation Kit (MoBio), DNA Isolation Kit for Cells and Tissues (Roche), and NucleoSpin™ Tissue (Macherey-Nagel). DNA yield was determined using a quantitative real-time PCR assay targeting IS*711 *that included an internal amplification control.

**Results:**

Kits of QIAGEN and Roche provided the highest amount of DNA, Macherey-Nagel and Peqlab products were intermediate whereas MoBio yielded the lowest amount of DNA. Differences were significant (p < 0.05) and of diagnostic relevance. Sample volume, elution volume, and processing time were also compared.

**Conclusions:**

We observed differences in DNA yield as high as two orders of magnitude for some samples between the best and the worst DNA extraction kits and inhibition was observed occasionally. This indicates that DNA purification may be more relevant than expected when the amount of DNA in tissue is very low.

## Background

Brucellosis is a 're-emerging' zoonosis with a worldwide distribution caused by small fastidious Gram negative bacteria of the genus *Brucella*. Patients present with non-specific signs and symptoms such as inconstant fevers, sweating, weakness, headaches, depression, weight loss, and muscle and body pain. The disease can become chronic with focal lesions which can affect various organs. The cultivation and identification of *Brucella *species with conventional microbiological techniques is time-consuming and hazardous and should be performed under BSL-3 conditions [1]. Real-time PCR assays allow for the rapid and specific detection of *Brucella *species, but the amount of bacteria in tissues and body fluids is frequently extremely low [2,3]. Therefore, DNA extraction is a critical step to avoid false negative PCR results. In general, blood and serum are regarded as valuable specimens, but biopsies of focal lesions are more relevant in chronic courses of the disease [4-6]. The material obtained with biopsies is usually scarce and can contain inhibitory components that make PCR diagnosis even more difficult. The performance of commercial kits for the preparation of DNA from serum has been evaluated by Queipo-Ortuno et al. [7], but no systematic study has been performed using clinical tissue specimens. Therefore, the aim of this study was to compare the performance of commercially available kits for the extraction of *Brucella *DNA from infected tissues.

## Methods

### Samples and tissue homogenization

Organ samples were obtained from two dromedaries from Dubai, United Arab Emirates (UAE), which were serological positive for brucellosis and had to be destroyed by UAE law (Ministerial degree of the UAE no. 132/2004). Both dromedaries were euthanized with T61 and samples were obtained from different organs (Tab. 1) for the isolation of *Brucella *organisms as described previously [8]. Culture proved generalized *Brucella melitensis *infections in both animals. Identification of the pathogen was performed using conventional microbiological techniques including species-specific AMOS PCR [9]. Six tissue samples of each animal were used to assess the performance of commercial DNA extraction kits. Initially, pieces of tissue (~5 g) were inactivated and preserved in formalin (10% v/v). Subsequently the specimens were washed twice in deionized water (10 ml), and incubated over night in 20 ml sterile physiological saline at 4°C. DNA extraction was done as described previously [10]. In brief, the specimens were cut into small pieces (~150 mg) and made up with PBS to 1 ml in a sampler tube. The tubes were shaken in a FastPrep^®^-24 Instrument (MP Biomedicals, Illkirch, France) at speed 5.0 for 20 s according to the manufacturer's instructions and placed on ice for further 20 s. This procedure was repeated five times. The sampler tubes were then centrifuged at 14.000 rpm for 20 min at 4°C. DNA was prepared from 50 μl aliquots of the supernatant.

**Table 1:** Mean concentration of *Brucella melitensis *DNA obtained with five purification kits from tissue samples of two dromedaries.

	QIAGEN		Peqlab		Macherey-Nagel		MoBio		Roche	
fg/μl	mean	SD	mean	SD	mean	SD	mean	SD	mean	SD
*D1452/06*										
Liver	8915	15150	186	17	76	9	neg		207	26
Spleen	10	2	5	2	101	21	neg*		402	20
Lung	225	66	72	11	216	55	71	23	2252	89
Right kidney	1377	19	73	27	315	24	101	19	757	53
Prescapular Ln	415	65	111	13	41	11	123	20	1338	355
Intestine	223	56	90	8	179	29	neg*		382	54
										
*D1469/06*										
Spleen	484	426	57	4	38	7	neg*		67	67
Right kidney	235	23	10	8	14	3	3	3	111	29
Spinal Cord	289	64	11	1	126	33	neg*		14*	12
Pancreas	229	57	61	10	179	34	75	15	88*	14
Aorta lymph node	208	42	4	2	238	41	neg		112	11
Mammary Ln	355	299	140	26	297	12	465	60	112	18

(Ln, lymph node; SD, standard deviation; neg, negative *Brucella *PCR assay; *, negative inhibition control).

### DNA purification methods

The five commercial kits used in this study are briefly described below along with the modifications introduced in each case to the manufacturer's instructions:

UltraClean™Tissue and Cells DNA Isolation Kit (MoBio): The protocol was followed to step 9. The elution (steps 10 to 12) was done using 100 μl TD3 buffer instead of 50 μl.

PeqGold™Tissue DNA Mini Kit (PeqLab): The protocol was followed from step 1 to step 7. Only the optional step 2 (RNase digestion) was not included. Elution was done using 100 μl elution buffer instead of 200 μl.

QIAampDNA™Mini Kit (QIAGEN): The protocol for tissue samples was followed including procedure 3a (without RNase treatment described in 3b) to step 8, but elution was done using 100 μl elution buffer.

DNA Isolation Kit for Cells and Tissues (Roche): The procedure for DNA isolation from 100 mg - 1 g tissue was followed as recommended by the manufacturer.

NucleoSpin™ Tissue (Macherey-Nagel): The protocol for genomic DNA purification from tissue was used as described by the manufacturer.

### Real-time PCR

The amount of DNA was determined in triplicates using a real-time PCR assay with hybridization probes targeting the insertion sequence IS*711*, which is present in 6-7 copies per genome in *Brucella abortus*, *melitensis*, and *suis*. An internal amplification control targeting bacteriophage lambda DNA was included to determine inhibitory effects of the sample matrix as described previously [11]. Primers and probes were designed based on alignment studies using the BLAST program and the sequence database of the National Centre for Biotechnology Information. The oligonucleotides used for the *Brucella *specific target were IS*711*_S TTGTCGATGCTATCGGCCTAC, IS*711*_R GGCAATGAAGGCCCTTAAGT, IS*711*_FL GAAGCTTGCGGACAGTCACCATAAT-fluorescein, and IS*711*_LC LightCycler-Red-640-GCCGGGTGTTGGCTTTATTCG-phosphate. The oligonucleotides used for the detection of the internal amplification control were: Lambda_F ATGCCACGTAAGCGAAACA, Lambda_R GCATAAACGAAGCAGTCGAGT, Lambda_FL.ss CACTTCCCGAATAAC-fluorescein, and Lambda_LC LightCycler-Red-705-CGGATATTTTTGATCTGACCGAAGCG-phosphate. Primers and probes were obtained from and designed in cooperation with TIB MOLBIOL (Berlin, Germany). A real-time hot-start PCR was performed with the LightCycler FastStart DNA Master Hybridisation Probes Kit™ (Roche Diagnostics, Mannheim, Germany) in a LightCycler Instrument™ (Roche Diagnostics, Mannheim, Germany). The 20 μl reaction mixture contained 3 mM MgCl_2_, 2 μl 10× LightCycler-FastStart Reaction Mix Hybridisation Probes, 0.5 μl of each primer (20 pmolμl^-1^), 0.5 μl of each hybridisation probe (8 pmolμl^-1^) and 2 μl template. No-template controls that contained 2 μl of PCR water instead of DNA and positive controls that contained DNA of *Brucella melitensis *were included in each run to detect any amplicon contamination or amplification failure. Reaction conditions were: 10 min at 95°C, 45 cycles of 10 s at 95°C, 10 s at 55°C and 15 s at 72°C. A melting curve analysis was performed after the last amplification cycle with 95°C for 0 s, 45°C for 30 s, and 95°C for 0 s. Temperature change rates were 20°Cs^-1 ^for all but the melting curve analysis, where the rate was 0.1°Cs^-1^. Data analyses were performed with LightCycler software version 5.32. The automated second derivative maximum method was used for standardized calculation of the threshold cycle number (C_T_). Readout of LightCycler-Red 640 values was performed in channel F2/F1 and readout of LightCycler-Red 705 values was performed in channel F3/F2. Cycle threshold values were used to calculate the amount of DNA that was prepared from the tissue samples. A positive result was assigned when at least two of the triplicate reactions gave a C_T _value < 40 and the values were used for further calculations. In order to generate a standard curve for the quantification of *Brucella *DNA the concentration of purified *B. melitensis *DNA was determined spectrophotometrically using a NanoDrop ND-1000 UV-Vis spectrophotometer (Nano-Drop Technologies, Wilmington, DE, USA). This quantified DNA was used for serial dilutions with PCR-grade water. Based on the full bacterial genome sequence of *B. melitensis *published in GenBank (accession numbers NC_003317 and NC_003318), it was determined that each *Brucella *genome copy (i.e., genome equivalent [GE]) amounts to 3.38 fg according to the following equation: GE (fg) = number of base pairs per GE × 618 g mol^-1 ^× 10^15^/6.023 × 10^23 ^mol^-1 ^(Avogadro constant).

### Statistics

Statistical analysis was performed using STATISTICA 7.1 (StatSoft Inc., Tulsa, OK 74104, USA) and Microsoft^® ^Office Excel 2003 (11.8105.8107) SP2. Significant differences between group values were determined by one-way ANOVA at p ≤ 0.05. One-way ANOVA assumes that the variances of the groups are all equal. Therefore a test for homogeneity of variances was performed (Levene test, data not shown). If the significance value exceeds 0.05, the assumption that the variances are equal is justified. In cases where differences among group means exist, post hoc range tests and pair wise multiple comparisons were performed (data not shown). For one-way ANOVA post hoc tests the Scheffe test was used if equal variances were shown. If equal variances could not be assumed the Dunnett's T3 test was used. A probit analysis to determine the limit of detection was performed using PriProbit^® ^10 version 1.63 designed by Dr. Masayuki Sakuma (Kyoto University, Kyoto, Japan, http://bru.gmprc.ksu.edu/proj/priprobit/index.asp). The statistical significance was set at a level of p = 0.05. The linear range of the insertion sequence IS*711*-based real-time PCR was determined according to the approved Clinical and Laboratory Standards Institute (CLSI) guidelines for the evaluation of the linearity of quantitative measurement procedures [12]. Seven equally spaced concentrations were analyzed with eight replicates at each level. Based on a polynomial method, two alternative statistical models (linear and nonlinear) were assessed relative to which is most likely to be true (1st-, 2nd- and 3rd- order least-squares regression). When a nonlinear polynomial fits the data better, it was assessed if the difference between the best-fitting nonlinear and linear polynomial is less than the amount of allowable bias for the method. If necessary, corrections (shortening of the linear range) were done and 1st-, 2nd- and 3rd- order least-squares regression was conducted again. The linear range was determined, when the linear model fitted best. Based on a generated standard curve (data not shown) the determined C_T _values of each sample were converted to quantitative results by using the logarithmic transformed function y = -1.8573Ln(x) + 41.18 with y = generated C_T _value and x = unknown DNA concentration.

## Results

The detection limit of the real-time PCR assay was 2.35 genome equivalents per PCR reaction with a probability of 95% as determined using Probit analyses. The linear range was calculated to be 260 to 2.6 × 10^7 ^fg per reaction. In order to detect inhibitory effects, a real-time PCR assay was incorporated specifically detecting bacteriophage λ-DNA as described previously (Table 1) [10]. The melting points measured for IS*711 *and the bacteriophage λ-DNA target were at approximately 64°C and 50°C, respectively.

Comparison of DNA yield obtained with the kits tested in this study showed significant differences as determined by one-way ANOVA (p < 0.05). Kits of QIAGEN and Roche provided the highest amount of DNA (Table 1), Macherey-Nagel and Peqlab products were intermediate whereas MoBio yielded the lowest amount of DNA. We also observed differences in *Brucella melitensis *DNA content between organs. One-way ANOVA (p < 0.05) showed significant differences of the mean between the amount of DNA in pancreas and the right mammary lymph node of dromedary D1469/06. In animal D1452/06 we found a significantly higher bacterial concentration in the right kidney than in spleen tissue. Inhibition of the PCR and/or insufficient DNA yield was observed with liver, spleen, intestine, lymph node, and spinal cord tissue preparations using the MoBio kit leading to negative results. Preparations of spinal cord and pancreatic tissues extracted using the Roche kit showed inhibition of the internal amplification control and also low DNA yield. For the selection of a DNA extraction method several factors besides the recovery of DNA and the elimination of inhibitory substances have to be considered for practical and economic reasons. Table 2 displays sample volume, elution volume, and processing time for each extraction method. All kits can be handled easily, but the hands-on time ranges from 30 min to 3 hrs 30 min (Table 2).

**Table 2:** Characteristics of DNA extraction kits.

Company	MoBio (Dianova)	Roche	QIAGEN	Peqlab	Macherey Nagel
**Kit**	UltraClean™ Tissue and Cells DNA Isolation Kit	DNA Isolation Kit for Cells and Tissues	QIAamp™ DNA Mini Kit	PeqGOLD™ Tissue DNA Mini Kit	NucleoSpin™ Tissue
**Cat. number**	12334-250	11814770001	51306	12-3396-02	740952.250
**Number of preparations**	250	10	250	200	250
**Sample size [mg]**	1-25	400	25	30	1-25
**Elution volume [μL]**	50	1000	50-200	50-200	60-100
**Processing time (hrs:min)**	00:15	02:30	02:30	03:20	03:15

## Discussion and Conclusions

The diagnosis of brucellosis is challenging as culture of Brucellae and sero-conversion require weeks. Nucleic acid amplification methods might circumvent this diagnostic window, but the very low amount of DNA in tissue samples obviates reliable rapid detection using real-time or conventional PCR techniques. Real-time PCR assays are rapid, specific and have a very low limit of detection, when multi-copy gene targets are used. However, in our experience with clinical samples even highly efficient and sensitive real-time PCR assays frequently fail to detect bacterial DNA due to the extremely low number of bacteria in tissues. For this reason, the aim of this study was to evaluate commercially available DNA extraction kits that should provide high DNA yields and reduce inhibition to improve the results that can be obtained with real-time PCR assays. Based on our experience formalin should be eliminated as much as possible in order to detect even the smallest amounts of target DNA. Therefore, we regarded washing steps and long incubation in saline an indispensable prerequisite. We have decided to test samples from naturally infected animals, because this allowed us to test several organs in parallel and to obtain enough tissue to evaluate a panel of kits. Due to the small amount of bacteria in some samples the DNA yield was sometimes out of the linear range of this real-time PCR assay which reflects the situation under real clinical conditions. Except for one kit (MoBio) all kits yielded enough DNA to regularly provide positive results in real-time PCR assays. The QIAGEN kit provided the highest DNA yield in half of the samples. The Roche kit can be advantageous when relatively large amounts of tissue need to be processed. We have observed differences in DNA yield as high as two orders of magnitude for some samples between the best and the worst DNA extraction kits in this study. This indicates that DNA purification may be more relevant than expected, especially, when the amount of DNA in tissue is very low. Optional RNase steps were performed in none of the assays as the reagents were not supplied with the kits and RNA does not inhibit the PCR reactions. In order to provide identical amounts of DNA eluate the amount of elution buffer was adjusted to 100 μl for all kits. It cannot be excluded that these minor changes of the purification procedure have adversely influenced the DNA yield of certain kits. In the animals used for this study we observed significant differences in DNA content among different organs. General recommendations regarding favorable tissue samples cannot be given and results obtained from specimens of focal lesions can only be part of a polyphasic diagnostic approach. Further studies in a clinical setting are warranted to assess, whether the use of these DNA preparation methods will actually help to improve the clinical usefulness of real-time PCR assays for the detection of Brucellae in tissue.

## Competing interests

ME was employee of QIAGEN and subsequently of Roche during the study period. The other authors declare that they have no competing interests whatsoever.

## Authors' contributions

HT designed and coordinated the study, drafted the manuscript and participated in performing real-time PCR assays. MK designed the real-time PCR assay and helped to draft the manuscript. ME performed the statistical analyses and participated in study design and drafting of the manuscript. UW isolated and identified *Brucella *and helped to draft the manuscript. HCS was responsible for DNA isolation, participated in the design of the study and helped to draft the manuscript. SA, ES, and HN participated in the design of the study, the evaluation of the PCR assays and helped to draft the manuscript. All authors read and approved the final manuscript.

## Pre-publication history

The pre-publication history for this paper can be accessed here:

http://www.biomedcentral.com/1471-2334/10/100/prepub

## References

[B1] Al Dahouk S Tomaso H Nöckler K Neubauer H Frangoulidis D Laboratory-based diagnosis of brucellosis - a review of the literature. Part I: techniques for direct detection and identification of *Brucella *spp Clin Lab 2003 49 487 505 14572205

[B2] Al Dahouk S Nöckler K Scholz HC Pfeffer M Neubauer H Tomaso H Evaluation of genus-specific and species-specific real-time PCR assays for the identification of *Brucella *spp Clin Chem Lab Med 2007 45 1464 1470 10.1515/CCLM.2007.305 17970716

[B3] Bricker BJ PCR as a diagnostic tool for brucellosis Vet Microbiol 2002 90 435 446 10.1016/S0378-1135(02)00228-6 12414163

[B4] Leal-Klevezas DS Martínez-Vázquez IO López-Merino A Martínez-Soriano JP Single-step PCR for detection of *Brucella *spp. from blood and milk of infected animals J Clin Microbiol 1995 12 3087 3090 10.1128/jcm.33.12.3087-3090.1995 PMC228649 8586678

[B5] O'Leary S Sheahan M Sweeney T *Brucella abortus *detection by PCR assay in blood, milk and lymph tissue of serologically positive cows Res Vet Sci 2006 81 170 176 10.1016/j.rvsc.2005.12.001 16545848

[B6] Zerva L Bourantas K Mitka S Kansouzidou A Legakis NJ Serum is the preferred clinical specimen for diagnosis of human brucellosis by PCR J Clin Microbiol 2001 39 1661 1664 10.1128/JCM.39.4.1661-1664.2001 11283112 PMC87995

[B7] Queipo-Ortuño MI Tena F Colmenero JD Morata P Comparison of seven commercial DNA extraction kits for the recovery of *Brucella *DNA from spiked human serum samples using real-time PCR Eur J Clin Microbiol Infect Dis 2008 27 109 114 10.1007/s10096-007-0409-y 17973130

[B8] Wernery U Kinne J Joseph M Johnson B Nagy P Where do Brucella organisms hide in serologically positive lactating dromedaries? Proc Int Camel Conf 16-17th February 2007. Bikaner, Rajasthan, INDIA 68 70

[B9] Bricker BJ Halling SM Differentiation of *Brucella abortus *bv. 1, 2, and 4, *Brucella melitensis*, *Brucella ovis*, and *Brucella suis *bv. 1 by PCR J Clin Microbiol 1994 11 2660 2666 10.1128/jcm.32.11.2660-2666.1994 PMC264138 7852552

[B10] Neubauer H Meyer H Prior J Aleksic S Hensel A Splettstoesser W A combination of different polymerase chain reaction (PCR) assays for the presumptive identification of *Yersinia pestis* J Vet Med B 2000 47 573 580 10.1046/j.1439-0450.2000.00384.x 11075545

[B11] Tomaso H Reisinger EC Al Dahouk S Frangoulidis D Rakin A Landt O Neubauer H Rapid detection of *Yersinia pestis *with multiplex real-time PCR assays using fluorescent hybridisation probes FEMS Immunol Med Microbiol 2003 38 117 26 10.1016/S0928-8244(03)00184-6 13129646

[B12] Clinical and Laboratory Standards Institute Evaluation of the linearity of quantitative measurement procedures: a statistical approach; approved guideline CLSI document EP6-A. Wayne, PA: CLSI 2003

